# Thinning and functionalization of few-layer graphene sheets by CF_4_ plasma treatment

**DOI:** 10.1186/1556-276X-7-268

**Published:** 2012-05-24

**Authors:** Chao Shen, Gaoshan Huang, Yingchun Cheng, Ronggen Cao, Fei Ding, Udo Schwingenschlögl, Yongfeng Mei

**Affiliations:** 1 Department of Materials Science, Fudan University, Shanghai, 200433, People's Republic of China; 2 PSE Division, King Abdullah University of Science and Technology (KAUST), Thuwal, 23955-6900, Kingdom of Saudi Arabia; 3 IBM Research − Zürich, Säumerstrasse 4, Rüschlikon, CH-8803, Switzerland

**Keywords:** Graphene sheets, Plasma, Thinning, Functionalization, Raman spectroscopy, Density functional theory, Dirac cone splitting

## Abstract

**Abstract:**

Structural changes of few-layer graphene sheets induced by CF_4_ plasma treatment are studied by optical microscopy and Raman spectroscopy, together with theoretical simulation. Experimental results suggest a thickness reduction of few-layer graphene sheets subjected to prolonged CF_4_ plasma treatment while plasma treatment with short time only leads to fluorine functionalization on the surface layer by formation of covalent bonds. Raman spectra reveal an increase in disorder by physical disruption of the graphene lattice as well as functionalization during the plasma treatment. The F/CF_3_ adsorption and the lattice distortion produced are proved by theoretical simulation using density functional theory, which also predicts p-type doping and Dirac cone splitting in CF_4_ plasma-treated graphene sheets that may have potential in future graphene-based micro/nanodevices.

**PACS:**

81.05.ue; 73.22.Pr; 52.40.Hf.

## Background

Graphene is one layer of C atoms, arranged in a hexagonal lattice [1-3]. Since it was first produced by mechanical exfoliation in 2004 [4], graphene has been studied both theoretically and experimentally, and demonstrates highly attractive properties [5-10]. Especially, the Dirac equation predicts that unique electronic properties should arise from the hexagonal honeycomb lattice structure, making the electrons behave as massless relativistic fermions [1,5,9]. The corresponding high mobility and velocity have great potential in future electronics [11,12]. However, several problems need to be solved before it can be ultimately employed in practical applications. For instance, the zero bandgap as well as bad wettability of graphene might cause problems in device fabrication [13-15]. Under such circumstances, plasma treatment is considered to be one of the tricks to overcome the difficulties. The corresponding surface functionalization changes not only the surface status, but also the structure of graphene sheets [16,17]. Previous researches have already demonstrated that oxygen plasma has the ability to tune the properties of graphene sheets [14,17]. On the other hand, fluorine plasma, which may provide additional advantages [16,18], is seldom experimentally investigated in detail, although a strong p-doping behavior was predicted [19,20]. In this work, structural changes of few-layer graphene sheets induced by CF_4_ plasma treatment are studied by optical microscopy and Raman spectroscopy. Our results suggest an obvious thickness reduction effect in few-layer graphene sheets treated with CF_4_ plasma as well as surface fluorine functionalization by formation of covalent bonds between the top graphene layer and the ions. The produced disorder in graphene lattice is well reflected in the Raman spectra, and the corresponding mechanism is studied theoretically. The results presented in this work provide a possible direction to obtain giant single-layer graphene sheets with necessary surface functionalization to realize the p-type doping level and to open the Dirac cone for future graphene-based micro/nanodevices.

## Methods

The few-layer graphene sheets used in the current study were produced by mechanical exfoliation from highly ordered pyrolytic graphite and transferred on a silicon substrate covered with 300-nm SiO_2_. The samples were then cleaned by ultrasonication in acetone for 30 s to remove the residual of the scotch tape and unattached graphite pieces. The CF_4_ plasma treatment was carried out in the reaction chamber of a Jupiter III reactive-ion etching setup. The samples were exposed to 0.8-Torr CF_4_ under radio-frequency plasma (20 W) with different times ranging from 1 to 5 s with a step of 1 s. In our experiment, the number of layers in the few-layer graphene sheets can be estimated by a combination of optical microscopy and Raman spectroscopy. Raman spectra were taken on a Renishaw inVia micro-Raman spectrometer with the 514-nm line of an Ar^+^ laser as light source (Shanghai, China). All the measurements were carried out at room temperature.

## Results and discussion

**Figure 1:**
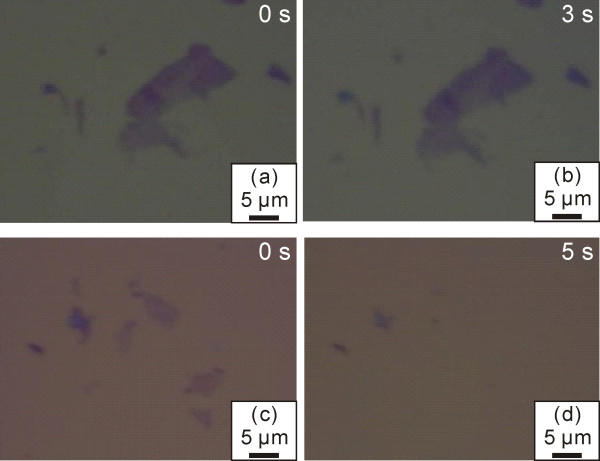
**Optical images of samples before and after CF**_**4**_**plasma treatment.** ( **a**, **c**) Optical images demonstrating the morphologies of as-prepared samples before CF_4_ plasma treatment. (**b**) Optical image of the sample in (a) after 3 s of CF_4_ plasma treatment. (**d**) Optical image of the sample in (c) after 5 s of CF_4_ plasma treatment.

As we know, morphology obtained by optical microscopy is a direct and convenient way to identify the few-layer graphene sheets. Optical images in Figure 1 demonstrate the surface morphologies of the samples before and after CF_4_ plasma treatment of different times. For a short plasma treatment (e.g., 2 s, see Figure 1a,b), no obvious change can be noticed at least in the optical images. However, with prolonged plasma treatment of up to 5 s, a thinning effect is obvious as the color contrast of the sample surface changes significantly. One can see in Figure 1c,d that dark purple regions appear lighter after treatment and some thin sheets can hardly be recorded by the camera (although they can be observed through eyepiece lens). During the process of plasma treatment, ions produced by an electromagnetic field attacked the sample surface and reacted with it. Therefore, the top layer of the sheet can be removed in longer treatment, which suggests an accumulation effect of the ion etching for such a low plasma power of 20 W.

**Figure 2:**
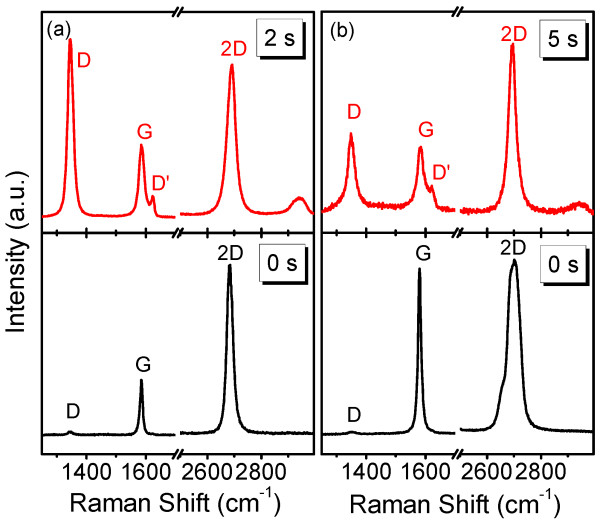
**Raman spectra of graphene sheets before and after CF**_**4**_**plasma treatment.** ( **a**) Raman spectra of a single-layer graphene sheet before (lower panel) and after (upper panel) 2 s of CF_4_ plasma treatment. (**b**) Raman spectra of a few-layer graphene sheet before (lower panel) and after (upper panel) 5 s of CF_4_ plasma treatment. A thinning effect is obvious.

However, a structural modification or surface functionalization may exist during CF_4_ plasma treatment [14,16,17], even though they cannot be detected by normal optical microscopy. Previous researches revealed that the structural modification may induce a shift of the phonon frequencies in graphene sheets which can be optically probed by Raman spectroscopy [10,21,22]. In order to guarantee the reproducibility of our experiment, the Raman signal was collected from the same spot with the assistance of optical microscopy. Figure 2a displays the Raman spectra of a graphene sheet before and after 2 s of CF_4_ plasma treatment, and, as previously discussed, the optical microscopy shows no change. The lower panel in Figure 2a is the Raman spectrum of the sample before plasma treatment, where two strong peaks are noticeable. The G band located at approximately 1,580 cm^−1^ is corresponding to optical E_2g_ phonons at the Brillouin zone center. The sharp 2D band, which is the overtone of the D peak and the sum of two phonons with opposite momentum, appears at approximately 2,680 cm^−1^[23]. The intensity ratio between the 2D and G bands (*I*_2D_/*I*_G_ = 3.2) and the small full width at half maximum (FWHM) of the 2D band (26 cm^−1^) indicate that the graphene sheet measured contains only one layer (i.e., single-layer graphene) [14,23]. Besides, a weak D band is detected at approximately 1,350 cm^−1^ and is connected to transverse optical phonons near the K point which require defects or lattice disorder (e.g., non-*sp*^2^ composition) for their activation via an inter-valley double-resonance Raman process [24]. Therefore, the weak D band in the sample before plasma treatment suggests that the single-layer graphene sheet in our experiment is of high quality. It is interesting to note that obvious changes exist in the Raman spectra of the sample after 2 s of plasma treatment (as shown in the upper panel of Figure 2a): (1) The D peak at approximately 1,350 cm^−1^ is remarkably intensified, suggesting an introduction of lattice disorder in the graphene sheet [24]; (2) the G peak is broadened and a shoulder D' band arises, which originates from intra-valley resonant Raman scattering [24,25]; (3) a new band occurs at approximately 2,941 cm^−1^ which is believed to be a combination of D and D' bands [17] or G and D bands [26,27]. These features in the Raman spectra are reported in the graphene sheet treated with oxygen plasma [14] and are the symbols of surface functionalization [14,16]. However, if the plasma treatment is elongated, the few-layer graphene sheet can be thinned and the evolution of Raman spectra becomes complicated. In our experiment, we found that single-layer graphene cannot survive the 5 s of CF_4_ plasma treatment. Thus, the Raman measurement was carried out on a few-layer graphene sheet which contains more than one layer before plasma treatment. The obtained spectrum was plotted in the lower panel of Figure 2b. The FWHM of the 2D peak is broadened to 53 cm^−1^ and the ratio of *I*_2D_/*I*_G_ is 1.1, elucidating that this sheet consists of a few layers. After 5 s of plasma treatment, the Raman spectrum in the upper panel of Figure 2b shows that the FWHM of the 2D band is reduced to 32 cm^−1^ and the ratio of *I*_2D_/*I*_G_ is increased to 2.5. The spectral evolution is different from that in 2 s of plasma treatment and indicates that the few-layer graphene layer is thinned to one or two layers [14,23,28], as can be observed by optical microscopy.

The evolution of the D band is the most important concern of many researches. The increase of its intensity is generally considered to be evidence that lattice disorder exists in the graphene sheet [14]. An interesting phenomenon arises when we look close to the intensity of the D band in a few-layer graphene sheet subjected to CF_4_ plasma for different times. The relative intensity of the D band (*I*_D_/*I*_G_) in the sample treated for 5 s is smaller than that for 2 s, implying that the disorder is even remarkable for shorter treatment. To understand this peculiar behavior, we must first make clear the mechanism of disorder production during plasma treatment. The disorder and the corresponding emergence of D and D' bands in our samples may arise from two processes: (1) physically, the order of pristine C atoms in graphene is disrupted by the CF_4_ plasma and some of the C atoms may be sputtered out, which is named as ion bombardment effect [29,30]; and (2) chemically, covalent bonds of fluorine-related species to the graphene lattice form during plasma treatment, leading to corresponding surface modification and functionalization. Both processes contribute to the increase of the disorder. As for the chemical process, the bond energies need to be considered. It was disclosed that the C-C bond in graphene owns higher bond energy (607 kJ/mol) than the C-F bond (485 kJ/mol). Thus, the C-C bond can be hardly broken, and hence CF_n_ (*n* = 1 to 3) or F species were only adsorbed on the top graphene layer by formation of covalent bonds, which will become saturated because the number of available active C atoms decreases with time. It is worth noting that the formation of covalent bonds can be evidenced by the upshift of 2D peak from 2,685 to 2,691 cm^−1^[31]. One may infer that the few-layer graphene sheet subjected to CF_4_ plasma treatment therefore possesses disorder features from both the physical and chemical interactions. The chemical interaction takes place only on the topmost layer, while the physical interaction accumulated with time and a long treatment can remove the top layer (i.e., thinning effect, as is reflected in the optical microscopy and Raman spectroscopy), exposing the beneath layer. The emergence and intensification of the D and D' bands in the sample subjected to 5 s of treatment thus originates from the disorder created in this new top layer and should increase gradually. Consequently, the disorder probed by Raman spectroscopy is even smaller in the sample treated by CF_4_ plasma for 5 s than in the sample treated for 2 s (see Figure 2a,b).

**Figure 3:**
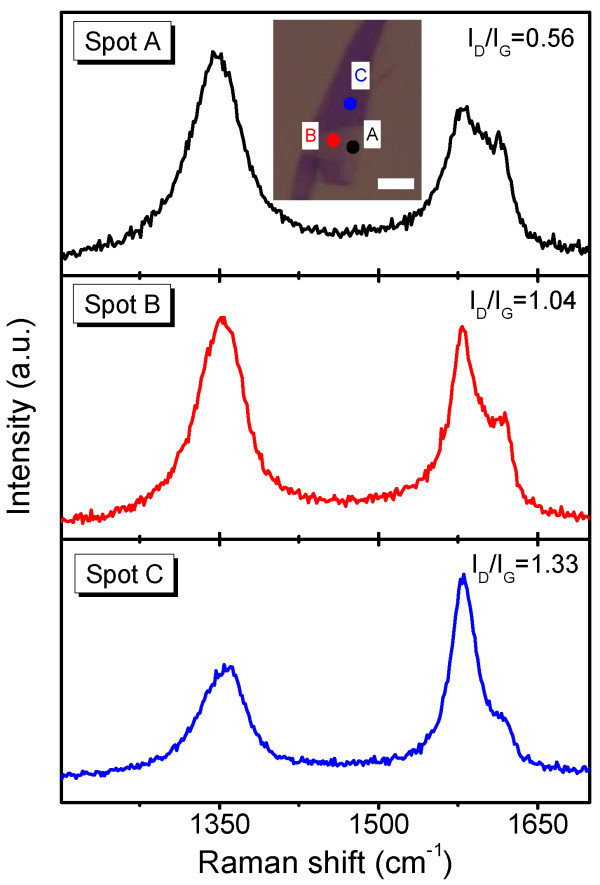
**Raman spectra of plasma-thinned few-layer graphene sheets with different thicknesses.** The three spectra were collected from three different spots respectively. The positions of the three spots are labeled in the inset optical image of a sample subjected to 5 s of CF_4_ plasma treatment. Scale bar, 5 μm.

Since the intensity of the D band reflects the disorder in the top layer of the few-layer graphene sheet, the relative intensity of the D band may give us a clue about the thickness of the sample. To prove this, we measured Raman spectra of plasma-thinned few-layer graphene sheets with different thicknesses. The three spectra in Figure 3 were respectively collected from three spots labeled in the inset optical image. The color contrast among the three spots indicates that their thicknesses are rather different and increase from A to C, while none of them consist a single layer. One can see that the *I*_D_/*I*_G_ is obviously relevant to the thickness of few-layer graphene sheet. For a thicker sheet, the weight of the topmost layer with remarkable disorder is smaller, and thus *I*_D_/*I*_G_ should be correspondingly smaller. Therefore, the ration of *I*_D_/*I*_G_ proves to be an effective Raman factor to compare the thicknesses of plasma-treated few-layer graphene sheets. Therefore, the *I*_D_/*I*_G_ proves to be another effective factor to estimate the thicknesses of plasma-treated few-layer graphene sheets other than *I*_2D_/*I*_G_.

**Figure 4:**
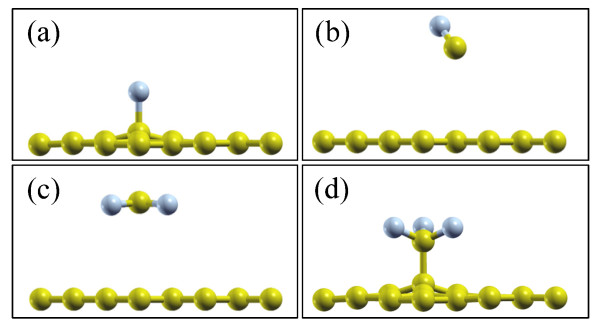
**Atomic structures of (a) F, (b) CF, (c) CF**_**2**_**, and (d) CF**_**3**_**ions on graphene sheets.** C and F atoms are indicated by yellow and gray balls, respectively. Theoretical simulation indicates that CF and CF_2_ can hardly be adsorbed.

Although a detailed experimental investigation is so far difficult, we managed to simulate the ion adsorption during the plasma treatment by employing density functional theory and the generalized gradient approximation of the exchange correlation functional with ultrasoft pseudopotentials [32,33] to reveal the disorder produced by fluorine functionalization and the underneath mechanism. A high cutoff energy of 800 eV and a k-point sampling with an 8 × 8 × 1 mesh are employed to achieve high accuracy in the calculations. Structural optimization is carried out on all systems until the residual forces are converged to 0.003 eV/Å. In order to avoid any drawback of the periodic boundary conditions, an over 20-Å-thick vacuum layer is included. We estimate that in the plasma, there are F, CF, CF_2_, and CF_3_ ions. The energy barrier is calculated by the climbing-image nudged elastic band method [34], which enables us to find the minimum energy path between the given initial and final states of a transition. By calculating the structures of ions on the graphene sheet (see Figure 4), we find that CF and CF_2_ can hardly be adsorbed, forming covalent bonds, while F and CF_3_ ions can be adsorbed. The corresponding adsorption energies are 2.2 and 0.4 eV, respectively. The different absorption behaviors can be understood as follows: Because of the *sp*^3^ hybridization nature for C atoms in CF, CF_2_, and CF_3_, there are two un-bonded electrons in CF_2_ and only one in CF_3_, while the C atoms in graphene are *sp*^2^ hybridized and there is only one un-bonded electron for each C atom. Thus, it is easier for CF_3_ to be adsorbed on top of the C atom via a covalent bond (see Figure 4d). However, it is difficult for CF_2_ to form two covalent bonds with two nearby C atoms in the graphene sheet. We checked different absorption configurations by structure relaxation and find that for all cases, the CF_2_ will leave away from the graphene, as shown in Figure 4c. For different artificial absorption configurations, there would be large strain/stress around the absorption site, indicating that the difficulty of forming two covalent bonds between CF_2_ and graphene is mainly due to the large strain/stress. A similar situation is also noted in the CF case. In addition, the calculated energy barrier for the CF_3_ adsorbed on the graphene surface is only 0.04 eV, which demonstrates that the CF_3_ adsorption is energetically favorable. Due to the existence of covalent bonds between F/CF_3_ and C on the graphene sheet, there is distortion around the adsorption position, which gives birth to the appearance of D and D' bands in the Raman spectra of the plasma-treated samples.

**Figure 5:**
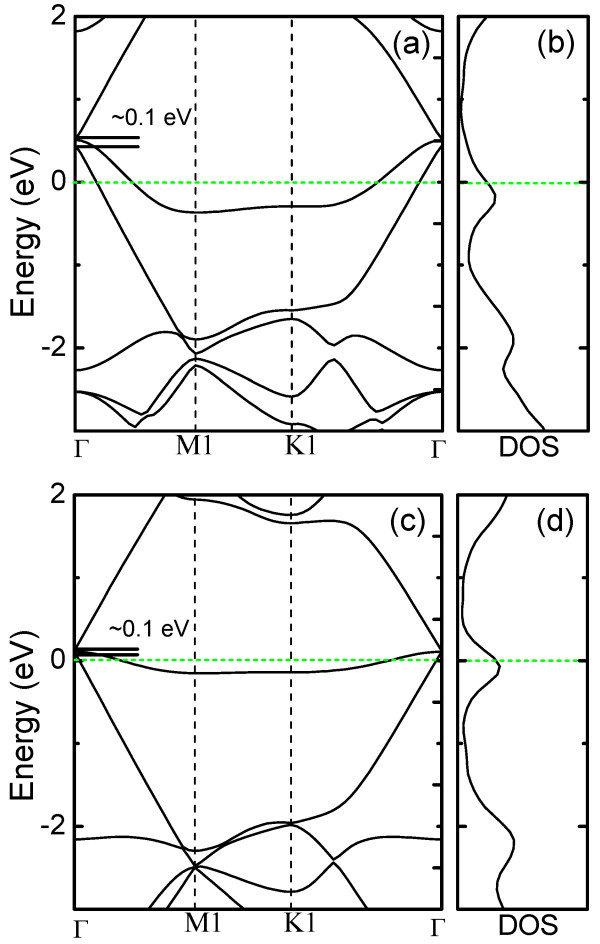
**Band structures and densities of states of F- and CF**_**3**_**-functionalized graphene sheets.** ( **a**) Band structure and ( **b**) density of states of a F-functionalized graphene sheet. ( **c**) Band structure and ( **d**) density of states of a CF_3_-functionalized graphene sheet. The Fermi level is set at 0 eV.

Here, we would like to briefly discuss the potential applications of this fluorine functionalization. We calculated the band structures and densities of states of graphene sheets fluorinated with F and CF_3_ with coverage of 5.6 %, as shown in Figure 5. Due to the Brillouin zone folding [20], the Dirac point is located at the Γ point in the electronic band structure. It is worth noting that due to the symmetry breaking, the Dirac cone splits with a small gap (approximately 0.1 eV). Moreover, the Dirac cone shifts to 0.5/0.1 eV above the Fermi level after F/CF_3_ functionalization due to electron transfer from the C atoms in the graphene sheet to F/CF_3_, indicating that the graphene sheets can be p-type doped by fluorinating with both F and CF_3_. However, it can be found that F is much more efficient to introduce p-type doping in graphene sheets than CF_3_. In addition, the adsorption of the F/CF_3_ on graphene sheets also introduces pinning states around the Fermi levels, as demonstrated in Figure 5. Detailed experimental verification is required in the future, while the presented calculation has already proven the application potentials of graphene sheets functionalized by CF_4_ plasma in micro/nanoelectronics.

## Conclusion

In conclusion, few-layer graphene sheets were prepared by mechanical exfoliation and the structural evolution during CF_4_ plasma treatment was studied in detail by optical microscopy and Raman spectroscopy. The experimental results indicate a thickness reduction under prolonged plasma treatment while short treatment leads only to fluorine functionalization on the surface layer. The combination of both physical and chemical reactions in the plasma treatment leads to structural modifications which can be well probed in Raman spectra. Theoretical simulation suggests a F/CF_3_ functionalization by formation of covalent bonds and also predicts a corresponding p-type doping and Dirac cone opening after the F/CF_3_ adsorption. Although further characterizations are needed to evaluate the electronic properties of treated samples, the current work of thinning and functionalizing few-layer graphene sheets by CF_4_ plasma under control represents an integrative pathway to industrial fabrication of graphene-based micro/nanodevices.

## Competing interests

The authors declare that they have no competing interests.

## Authors' contributions

CS, GH, FD, and YM designed the study. CS performed the experiments with help from RC. YC and US carried out the theoretical study. CS, GH, YC, and YM contributed in drafting the manuscript. All the authors took part in the discussion of the results, and edited and approved the manuscript.
